# GeneMesh: a web-based microarray analysis tool for relating differentially expressed genes to MeSH terms

**DOI:** 10.1186/1471-2105-11-166

**Published:** 2010-04-01

**Authors:** Saurin D Jani, Gary L Argraves, Jeremy L Barth, W Scott Argraves

**Affiliations:** 1 Department of Regenerative Medicine and Cell Biology, Medical University of South Carolina, Charleston, SC 29425 USA; 2 Array Genetics, Inc., Newtown, CT 06482, USA

## Abstract

**Background:**

An important objective of DNA microarray-based gene expression experimentation is determining inter-relationships that exist between differentially expressed genes and biological processes, molecular functions, cellular components, signaling pathways, physiologic processes and diseases.

**Results:**

Here we describe GeneMesh, a web-based program that facilitates analysis of DNA microarray gene expression data. GeneMesh relates genes in a query set to categories available in the Medical Subject Headings (MeSH) hierarchical index. The interface enables hypothesis driven relational analysis to a specific MeSH subcategory (e.g., Cardiovascular System, Genetic Processes, Immune System Diseases etc.) or unbiased relational analysis to broader MeSH categories (e.g., Anatomy, Biological Sciences, Disease etc.). Genes found associated with a given MeSH category are dynamically linked to facilitate tabular and graphical depiction of Entrez Gene information, Gene Ontology information, KEGG metabolic pathway diagrams and intermolecular interaction information. Expression intensity values of groups of genes that cluster in relation to a given MeSH category, gene ontology or pathway can be displayed as heat maps of Z score-normalized values. GeneMesh operates on gene expression data derived from a number of commercial microarray platforms including Affymetrix, Agilent and Illumina.

**Conclusions:**

GeneMesh is a versatile web-based tool for testing and developing new hypotheses through relating genes in a query set (e.g., differentially expressed genes from a DNA microarray experiment) to descriptors making up the hierarchical structure of the National Library of Medicine controlled vocabulary thesaurus, MeSH. The system further enhances the discovery process by providing links between sets of genes associated with a given MeSH category to a rich set of html linked tabular and graphic information including Entrez Gene summaries, gene ontologies, intermolecular interactions, overlays of genes onto KEGG pathway diagrams and heatmaps of expression intensity values. GeneMesh is freely available online at http://proteogenomics.musc.edu/genemesh/.

## Background

DNA microarray analysis typically involves determining inter-relationships that exist between differentially expressed genes and biological processes or categories. Such information is available in the form of gene ontologies, which describe gene products in terms of their associated biological processes, cellular components and molecular function (i.e., Gene Ontology [1]). Although employed by numerous computational algorithms, these databases lack the full breadth of information contained in the literature. To address this problem, approaches are emerging that utilize the body of information contained in the Medical Subject Headings (MeSH) [2] of the U.S. National Library of Medicine (NLM) for interpreting DNA microarray data [3,4]. MeSH is a hierarchically structured compilation of nearly 25,000 descriptors that include broad and specific headings/categories. MeSH categories are populated by articles from 4,800 of the world's leading journals indexed in the MEDLINE/PubMed database. The National Center for Biotechnology Information (NCBI) offers a MeSH database that provides links to all PubMed citations that correspond to a given MeSH term. Furthermore, NCBI makes available the Gene Links display feature that provides access to the genes (GeneIDs) mentioned in all PubMed articles.

Our motivation for this project was to develop a web-based system that would facilitate analysis of DNA microarray gene expression data so as to detect relationships between groups of differentially expressed genes and the categories available in the MeSH hierarchical index. Importantly, the system needed to analyze the entirety of genes in an expression dataset, not simply query the MeSH hierarchical index using single genes. Ideally, the system would facilitate hypothesis testing by determining if an experimental stimulus elicits specific effects on genes belonging to a particular MeSH category. Furthermore, the system would display heatmaps of expression intensity values in order to show the way in which multiple genes, associated with a particular MeSH category, behaved in response to the experimental stimulus. Finally, the system would execute rapid linking of genes to their meta information, including the information contained in Entrez Gene, Gene Ontology, KEGG pathway and intermolecular interaction databases. Here we report on the development of a system that meets these specifications.

## Implementation

### Program Design

The GeneMesh user interface is written in both HTML and a Web 2.0 technology, jQuery [5]. The backend is written in Perl [6] and PHP [7] and uses a MySQL Database (version 5.0.45) [8] operating on an Apache [9] web server running a Linux Fedora 7 [10] operating system. The GeneMesh Database is populated through a multi-step process. First, a search algorithm performs iterative web-based queries to collect all PubMed IDs linked to individual MeSH terms contained in the NLM MeSH Trees file. Only those MeSH terms having three or more parent nodes are employed in this process. Next, Gene IDs associated with the collected PubMed IDs are obtained from NCBI (file gene2pubmed.gz), and then the Gene ID, PubMed ID and MeSH associations are stored in a MySQL (Sun Microsystems) database (i.e., GeneMesh Database). For each MeSH category, the PubMed IDs and Gene IDs are tabulated and used to calculate the average number of citations per unique Gene ID. Additional information stored in the database includes gene ontology information for each Gene ID (obtained from the NCBI file, gene2go.gz), HomoloGene IDs (obtained from the NCBI file, homologene.data) and GeneMesh Database statistics (e.g., total number of unique MeSH terms associated with GeneIDs, number of top-level MeSH categories populated with GeneIDs and the total number of HomoloGene IDs represented). This information can be viewed using the html link, DB Stats.

### Input

GeneMesh is configured to analyze DNA microarray gene expression data derived from a number of commercial microarray platforms, including Affymetrix, Agilent and Illumina. Microarray data is input as a Comma Separated Value (CSV) file containing the probe identifiers and log base 2 intensity values. The program operates on subsets of expression data (e.g., a set of differentially expressed genes having < 500 genes), but many features also operate on an entire microarray data set.

Major input features of the program enable users to identify genes in a data set that associate with a specified MeSH category (e.g., major MeSH category: Disease; sub category: Congenital, Hereditary, and Neonatal Diseases and Abnormalities) or word/phrase (e.g., angiogenesis, DiGeorge syndrome, extracellular matrix or stem cells). Users may employ a filter to govern the stringency of this identification process. For example, the analysis may be confined to GeneID/MeSH associations for which GeneIDs have citation counts that exceed the average for all GeneIDs in a given MeSH category; this is done by selecting '>Avg' from the pull down menu. Similarly, the analysis may be made more stringent by limiting the analysis to GeneID/MeSH associations for which GeneIDs have citation counts greater than one standard deviation above the mean; this is done by selecting the '>StDev' pull down. The user may also employ an unfiltered analysis in which all GeneID/MeSH associations are included; this is done by selecting 'Unfiltered' from the pull down menu.

## Results

**Figure 1:**
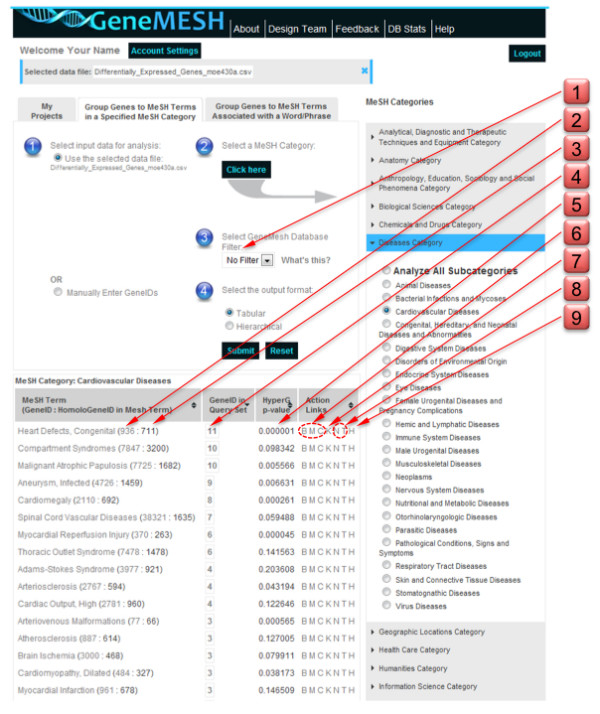
**Screenshot of the GeneMesh user interface**. Red tabs along the right margin highlight input and output action links including: 1, pulldown for selecting limits on inclusion of genes into MeSH categories based on citation statistics; 2, the total number of GeneIDs associated with the given MeSH term; 3, the total number of HomoloGene IDs associated with the given MeSH term; 4, the number of genes from the input set that match genes associated with the indicated MeSH term (clicking on the number links to Entrez Gene summaries for each of the genes); 5, the hypergeometric probability of genes associated with a given MeSH category (clicking on the arrow sorts output MeSH categories in ascending or descending order based on the magnitude of the hypergeometric p value); 6, the B, M or C action links provide gene ontology information; 7, the K action link links to KEGG pathway diagrams for genes associated with a given MeSH category; 8, links to protein interaction information displayed in a dynamically interactive network (N) or links to protein interaction information displayed in a tabular format (T); and 9, the H action link display a Heat map of Z score-normalized hybridization intensity values for the set of genes associated with a given MeSH category.

GeneMesh displays a table of all MeSH subcategories detected from the input DNA microarray data file (Figure 1). GeneIDs in each MeSH category are linked to information at NCBI Entrez Gene and a hypergeometric probability value [11] for the MeSH category is provided. The magnitude of the hypergeometric p value can be used to sort output MeSH categories in ascending or descending order. In addition to the automated parsing of input genes in relation to MeSH categories, the program will also associate genes from the input gene set with a specific user-entered search term, which may or may not be a MeSH term.

Through a series of action links, the program depicts genes associated with a given MeSH category in relation to Gene Ontology [1] information (i.e., biological processes, molecular functions, cellular components and pathways derived from the gene2go.gz file at NCBI Entrez Gene). Using the amCharts software bundle (amCharts.com) ontology information is displayed as animated pie charts with clickable slices that are linked to NCBI Entrez Gene information. Through another action link (designated K), GeneIDs associated with a given MeSH category are linked to Kyoto Encyclopedia of Genes and Genomes (KEGG) metabolic pathways and their diagrams [12]. An action link (designated VIV for view intensity values) permits viewing expression intensity values for a given gene name, gene symbol or probe ID in the input data set. This feature also allows heat maps to be generated for the selected genes.

The program will define intermolecular interactions known to exist between the protein products of genes associated with a given MeSH category or subcategory. Protein interaction information is displayed either in a tabular format or in a dynamically interactive network generated using the GUESS program http://graphexploration.cond.org/. The identified interactions are based on data contained in the file interactions.gz from NCBI ftp://ftp.ncbi.nlm.nih.gov/gene/GeneRIF. Interaction data contained in this file is derived from multiple sources including BIND, EcoCyc, BioGRID and HPRD (see ftp://ftp.ncbi.nlm.nih.gov/gene/GeneRIF/interaction_sources and GeneRIF.

GeneMesh produces heat maps of Z score-normalized hybridization intensity values of genes in a query set that are associated with a given MeSH category. Heat maps are generated using the heatmap.2 function in the R gplots package and probe pair annotations available through Bioconductor [13]. Dotplots are generated using the dotchart function in the R graphics package.

In addition to relating input genes to MeSH categories, the GeneMesh program also performs a number of useful upfront operations on input expression data including viewing of all genes in the data set with links to Entrez Gene summaries (using the 'V' action link), viewing of expression intensity values of genes in the data set (using the 'VIV' action link), grouping of genes in relation to gene ontologies (including biological processes, molecular functions and cellular components using the 'B', 'M' and 'C' action links, respectively), mapping of GeneIDs of individual genes or clusters of genes to KEGG pathway diagrams (using the 'K' action link) and generating heatmaps of expression intensities (using the 'H' action link).

GeneMesh has other useful query and browsing features that do not require input expression data. For example, if a user is interested in identifying genes associated with a particular process, the "*Group genes to Mesh terms associated with a word/phrase*" feature allows a user to input a term (e.g., asthma) and obtain a list of Mesh categories and their associated GeneIDs.

**Figure 2:**
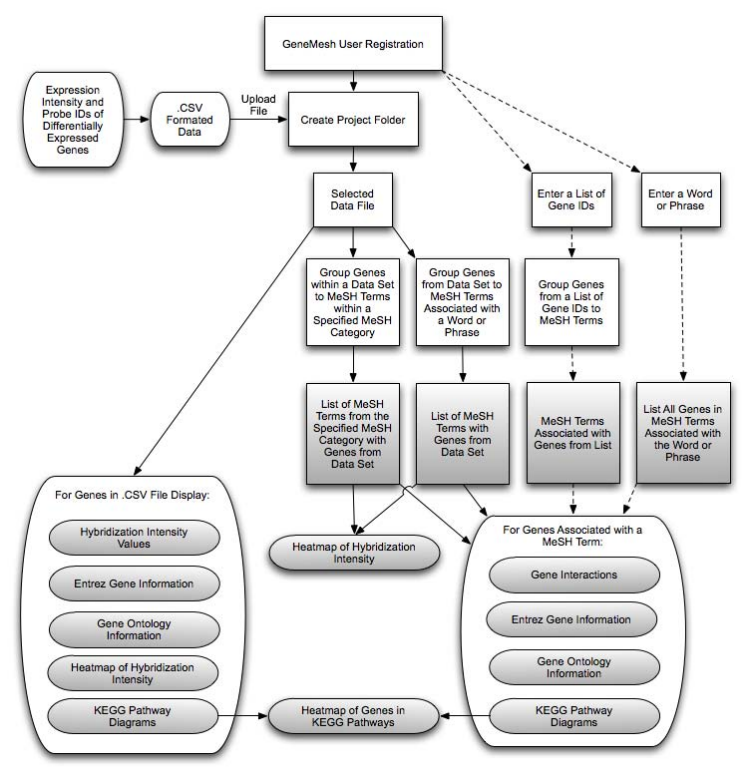
**Diagram of the flow of input and output information using the GeneMesh program**. After registration a user can upload a microarray dataset in the .CSV format and select a variety of action operations (boxed in the flowchart). Registered users may also enter a list of GeneIDs to obtain MeSH terms associated with genes in the list or they may enter a word or phrase and obtain associated MeSH terms and genes. Dotted lines in the flowchart connect operations that can be performed without uploading a data file. The various types of information outputs are shaded in grey.

A diagram of the flow of data into the GeneMesh program together with operation options and outputs is depicted in Figure 2. An example of a GeneMesh analysis of a group of differentially expressed genes is given in an Additional Material file (Additional File 1). The GeneMesh web site also provides access to tutorials, which are offered in both html and video formats.

## Conclusion

In this paper we describe a new DNA microarray data analysis tool, GeneMesh, which can accelerate discovery by identifying significant inter-relationships that exist between differentially expressed genes and MeSH categories. The fact that the user can specify that an analysis be limited to a specific subcategory (e.g., Cardiovascular diseases) or a broad category (e.g., Diseases) allows for biased and unbiased analyses. Furthermore, through the use of a robust set of action link features the user can quickly see how genes associated with a MeSH category relate to biological processes, molecular functions, cellular components and signaling pathways. Through access to heatmaps the user can readily see how the genes in any given MeSH categories or any related ontological category behave in response to the experimental stimuli used to generate the original differentially expressed gene data set.

A limited number of online tools can analyze gene(s) based on MeSH associations. These tools, such as Gendoo [14], MeSHer [15] and HuGE Navigator [16], have varying capabilities for data input, results browsing and significance analysis (Table 1). GeneMesh is distinct in its user interface (i.e., Web 2.0) and analysis features, combining the abilities to use gene identifiers as input data (as opposed to microarray platform-specific identifiers), permit upload of expression data for the gene set, and execute a significance analysis that can be adjusted for stringency. Furthermore, GeneMesh offers many unique options for browsing the analysis results in graphical or tabular format, either as a whole or partitioned according to other functional features.

**Table 1:** Outline of features for GeneMesh and other web tools with MeSH analysis capabilities.

Feature	GeneMesh	Gendoo	MeSHer	HuGE Navigator
Map gene(s) to MeSH categories^*a*^	Yes	Yes	Yes	Yes
Browse MeSH relationships^*b*^	Yes	Yes	Yes	Yes
Microarray platform independence^*c*^	Yes	Yes	-	Yes
Capable of analyzing a group of genes^*d*^	Yes	Yes	Yes	-
Significance analysis of MeSH categories^*e*^	Yes	Yes	Yes	-
Significance analysis threshold is adjustable^*f*^	Yes	-	Yes	-
Expression data upload^*g*^	Yes	-	-	-
Graphical display of expression data (e.g., heatmaps, tables)	Yes	-	-	-
MeSH output can be viewed by functional attributes (e.g., KEGG pathway, Gene Ontology, NCBI interaction)^*h*^	Yes	-	-	-
Web 2.0 design	Yes	-	-	-

^*a *^Software identifies links between MeSH categories and genes

^*b *^Software offers a browser feature to navigate through gene associations and/or the MeSH framework

^*c *^Software analyzes based on gene name, symbol or ID as opposed to a sequence identifier associated with a specific microarray platform

^*d *^Software can analyze a group of input genes as opposed to a single gene

^*e *^Software performs a statistical analysis to identify significant MeSH categories for the input gene set

^*f *^Threshold for significance analysis can be adjusted to govern stringency

^*g *^Software will upload expression data together with the gene set

^*h *^Output is presented to facilitate browsing of other functional attributes, including KEGG pathway, Gene Ontology, and NCBI interactions

## Authors' contributions

SJ and WSA conceived the original GeneMesh framework, JLB contributed ideas for functionality, and WSA subsequently oversaw programming by SJ. GLA wrote the code that performed web-based querying of the MeSH database to collect PubMed IDs that can be linked to individual MeSH terms contained in the NLM MeSH Trees file. SJ, JLB and WSA wrote the manuscript. All authors read and approved the final version of the manuscript.

## Availability and requirements

Project name: GeneMesh

Project homepage: http://proteogenomics.musc.edu/genemesh/

Operating system: User interface: Platform independent; Server side: Linux

Programming language: Perl, R, PHP, HTML and jQuery

Other requirements: Web browser (supporting JavaScript)

Any restrictions to use by non-academics: License needed

For reviewers of the software: To enable reviewers to test the GeneMesh software in a way that preserves their anonymity we have created a demo account that can be accessed at http://cbrc.musc.edu/homepage/jani/genemesh/index.html by entering the username 'demo@demo.com' and the password 'testgenemesh'.

## Supplementary Material

Additional file 1 **Supplementary information**. An example of a GeneMesh analysis conducted on a list of differentially expressed genes. Click here for file
