# Supplementary material for: GeneMesh: a web-based microarray analysis tool for relating differentially expressed genes to MeSH terms
Source: BMC Bioinformatics. 2010 Apr 1;11:166. doi: 10.1186/1471-2105-11-166 (PMC3212930; doi:10.1186/1471-2105-11-166)
Supplement: Additional file 1 — Supplementary information. An example of a GeneMesh analysis conducted on a list of differentially expressed genes. [file 1471-2105-11-166-S1.PDF]

Additional Material

Additional File 1: Supplementary information. An example of a GeneMesh analysis conducted on a list of differentially expressed genes.

A. GeneMesh identifies MeSH associations among a differentially expressed gene set.

Data input: 158 differentially expressed genes (not shown).

Select major MeSH category: Anatomy

Select Mesh subcategory: Cardiovascular System

Select GeneMesh Database Filter: >Avg

Select the output format: Tabular

Press Submit

Output: Figure 1

Perform list sorting: Based on hypergeometric p value

MeSH Category: Cardiovascular System

MeSH Term (GeneID : HomoloGeneID in Mesh Term)	GeneID in Query Set	HyperG p-value	Action Links
Myocytes, Cardiac (441 : 286)	8	0.000012	B M C K N T H
Brachiocephalic Trunk (2 : 2)	1	0.007436	B M C K N T H
Sinus of Valsalva (2 : 2)	1	0.007436	B M C K N T H
Capillaries (164 : 133)	3	0.012119	B M C K N T H
Aortic Valve (6 : 5)	1	0.018384	B M C K N T H
Aorta (116 : 83)	2	0.034908	B M C K N T H
Carotid Arteries (110 : 93)	2	0.042290	B M C K N T H
Aorta, Abdominal (14 : 12)	1	0.042989	B M C K N T H
Aorta, Thoracic (40 : 37)	1	0.120788	B M C K N T H
Pericytes (52 : 40)	1	0.129134	B M C K N T H
Tunica Media (46 : 42)	1	0.134586	B M C K N T H

Figure 1. Sample tabular output of genes from the query set having relationships to subcategories of the 'Cardiovascular System' MeSH category

B. Clustering of gene expression patterns from the 'Myocytes, Cardiac' MeSH subset shows that 7 genes are upregulated and 1 is downregulated in the experimental group.

From the output (***Figure 1***) select H Action Link to display a heat map of the 8 genes associated with the MeSH term "Myocytes, Cardiac".

Output: Figure 2

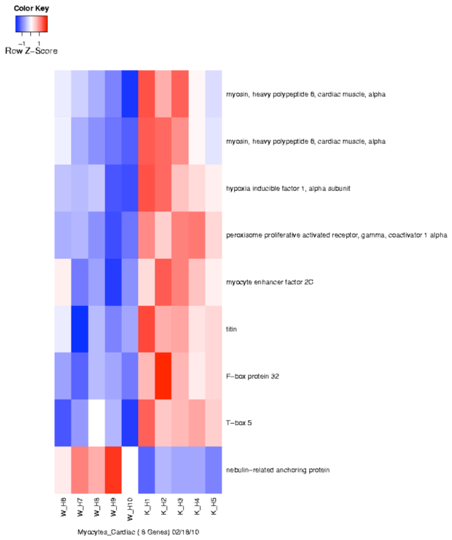

Figure 2. Sample heatmap showing the expression of each of the 8 genes in replicate control and experimental samples. Red, high; Blue, low.

C. Graphic display of gene ontologies for the differentially expressed genes associating with ‘Myocytes, Cardiac’ shows that several are transcription factors.

From the output (***Figure 1***) select M to display interactive pie chart of Gene Ontology Information: Molecular Function. The select light green pie slice corresponding to ‘’Transcription Factor Activity’.

Output: Figure 3

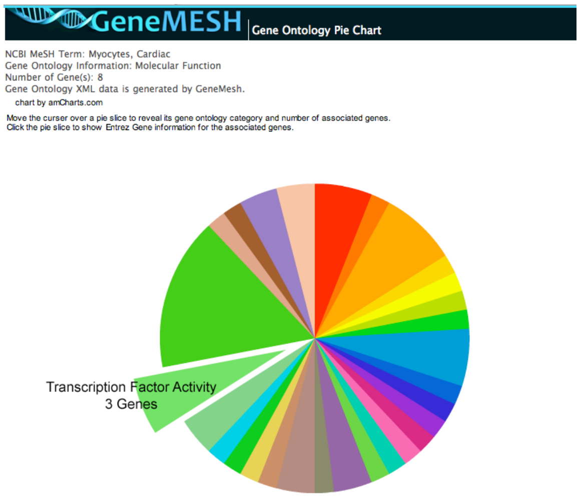

Figure 3. Sample interactive pie chart of the Gene Ontology Information (Molecular Function) related to the 8 genes associated with the MeSH term “Myocytes, Cardiac” shown in Figure 1.

D. The differentially expressed transcription factors within the ‘Myocytes, Cardiac’ MeSH subset group are Mef2c, Hif1α, and Tbx5.

From the output (***Figure 3***) click the yellow pie slice to obtain Entrez Gene information of genes associated with the gene ontology category represented by the light green pie slice.

Output: Figure 4

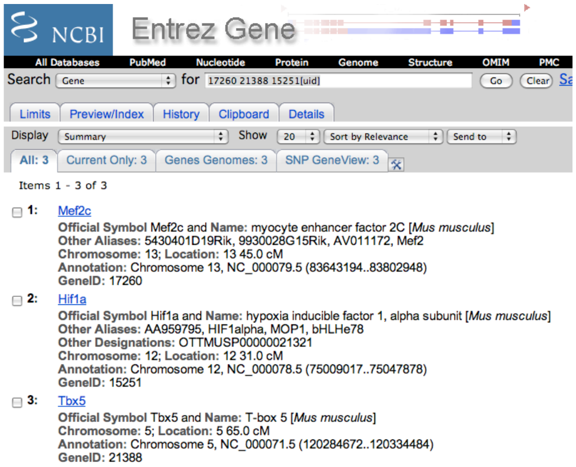

Figure 4. Sample Entrez Gene information associated with the genes in the ontology category (light green pie slice) selected in Figure 3.

E. Differentially expressed genes among the 'Myocytes, Cardiac' MeSH subset belong to several signaling cascade pathways.

From the output (***Figure 1***) select K Action Link to display KEGG pathways related with the 8 genes associated with the MeSH term "Myocytes, Cardiac".

Output: Figure 5

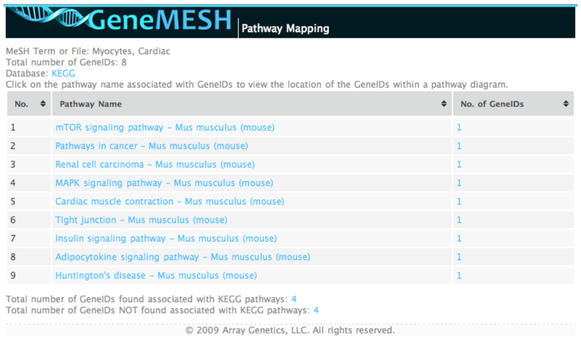

No.	Pathway Name	No. of GeneIDs
1	mTOR signaling pathway - Mus musculus (mouse)	1
2	Pathways in cancer - Mus musculus (mouse)	1
3	Renal cell carcinoma - Mus musculus (mouse)	1
4	MAPK signaling pathway - Mus musculus (mouse)	1
5	Cardiac muscle contraction - Mus musculus (mouse)	1
6	Tight junction - Mus musculus (mouse)	1
7	Insulin signaling pathway - Mus musculus (mouse)	1
8	Adipocytokine signaling pathway - Mus musculus (mouse)	1
9	Huntington's disease - Mus musculus (mouse)	1

Figure 5. Sample KEGG pathway mapping of the 8 genes associated with the MeSH term "Myocytes, Cardiac" shown in Figure 1.

C. Graphical display of pathways shows how Hif1α fits within the mTOR signaling pathway.

From the KEGG pathway mapping list (***Figure 5***) click the pathway named "mTOR signaling pathway" to display a KEGG pathway diagram with the related gene highlighted in red.

Output: Figure 6

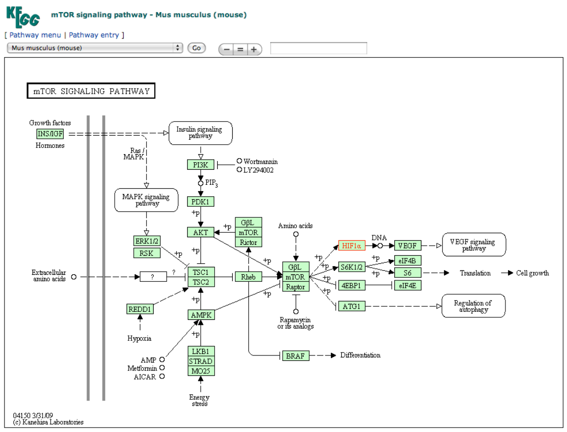

Figure 6. Sample KEGG pathway diagram in which genes associated with the MeSH term "Myocytes, Cardiac" and associated with the mTOR signaling pathway are highlighted in *red*.
